# Bacterial biofilm prevalence in dental unit waterlines: a systematic review and meta-analysis

**DOI:** 10.1186/s12903-023-02885-4

**Published:** 2023-03-18

**Authors:** Mojtaba Bayani, Kiyavash Raisolvaezin, Amir Almasi-Hashiani, Seyed Hamed Mirhoseini

**Affiliations:** 1 Department of Periodontics, School of Dentistry, Arak University of Medical Sciences, Arak, Iran; 2 Student Research Committee, Arak University of Medical Sciences, Arak, Iran; 3 Department of Epidemiology, School of Health, Arak University of Medical Sciences, Arak, Iran; 4 Department of Environmental Health Engineering, School of Health, Arak University of Medical Sciences, Arak, Iran

**Keywords:** Microbial Biofilm, Bacterial Biofilm, Contamination, Microbial Contamination, Dental unit, Dental unit waterline, Meta-Analysis

## Abstract

**Backgrounds:**

Numerous studies have shown that dental unit water lines (DUWLs) are often contaminated by a wide range of micro-organisms (bacteria, fungi, protozoa) and various prevalence have been reported for it in previous studies. Therefore, this review study aims to describe the prevalence of bacterial biofilm contamination of DUWLs.

**Methods:**

This is a systematic review and meta-analysis in which the related keywords in different international databases, including *Medline* (via *PubMed*) and *Scopus* were searched. The retrieved studies were screened and the required data were extracted from the included studies. Three standard methods including *American Dental Association* (ADA), *The Center for Disease Control and Prevention (CDC)* and contaminated > 100 CFU/ml(*C-100*) standards were used to assess the bacterial biofilm contamination of DUWLs. All studies that calculated the prevalence of bacterial biofilm contamination of DUWLs, and English full-text studies were included in the meta-analysis. Studies that did not have relevant data or used unusual laboratory methods were excluded. Methodological risk of bias was assessed by a related checklist and finally, the data were pooled by fixed or random-effect models.

**Results:**

Seven hundred and thirty-six studies were identified and screened and 26 related studies were included in the meta-analysis. The oldest included study was published in 1976 and the most recent study was published in 2020. According to the ADA, *CDC* and *C-100* standards, the prevalence of bacterial contamination was estimated to be 85.0% (*95% confidence interval (CI)*: 66.0–94.0%), 77.0% (*95%CI*: 66.0–85.0%) and 69.0% (*95%CI*: 67.0–71.0%), respectively. The prevalence of *Legionella Pneumophila* and *Pseudomonas Aeruginosa* in DUWLs was estimated to be 12.0% (*95%CI*: 10.0–14.0%) and 8.0% (*95%CI*: 2.0–24.0%), respectively.

**Conclusion:**

The results of this review study suggested a high prevalence of bacterial biofilm in DUWLs; therefore, the use of appropriate disinfecting protocol is recommended to reduce the prevalence of contamination and reduce the probable cross-infection.

**Supplementary Information:**

The online version contains supplementary material available at 10.1186/s12903-023-02885-4.

## Introduction

Dental units consist of a set of systems (e.g., air, water, and electricity source) as well as various equipment needed to perform various dental treatments [1, 2]. These units use water to cool instruments (including high-speed turbines, handpieces, etc.) and to rinse tooth surfaces during various operations [3]. This water is also used to rinse the patient's mouth. The dental unit water lines (DUWLs) are a complex network of narrow-bore plastic tubes, valves, and connectors that make water available to different parts of the unit. Numerous studies have shown that DUWLs are often contaminated by a wide range of micro-organisms (bacteria, fungi, protozoa) which originate from water supply sources [4–6].

Due to the conditions provided within DUWLs, microorganisms in the water can adhere to the inner surfaces of the tubes, form microcolonies, and eventually form a bacterial biofilm [7]. In the early 1960s, the first reports of the presence of large numbers of microorganisms in DUWLs were presented, and following that, the number of these reports increased in the following years [8]. The main water-related contaminants in dental units are of bacterial origin because bacteria are the predominant part of the microflora, although many studies have also shown the presence of fungi and protozoa [9, 10].

Microbial contamination of DUWLs originates primarily from the unit's water supply, which usually carries small amounts of microorganisms [1, 11, 12]. Despite the standards set for the water quality of the dental units, the formation of biofilms is an inevitable process, and microbial cells can be released into the water stream that eventually reaches the patient's mouth. Since the DUWLs are usually composed of several meters of plastic tubing with an inner diameter of about 1–2 mm, various factors (including different water flows in the center and sides of the walls or the laminar flow; stagnation of water in the intervals between the use of equipment; and also the absence of valves preventing the return of water, which prevent the return of fluids in the patient's mouth to the water pipes), cause the accumulation of microorganisms and provide conditions for biofilm formation [13, 14]. According to studies, *Pseudomonas aeruginosa* and *Legionella pneumophila* are the most common species of opportunistic pathogens found in the biofilms of the DUWLs, which have raised concerns about cross-infections, especially in immunocompromised individuals [2, 11, 15].

Both patients and dental personnel are exposed to contaminated water from waterways, either directly (sprinkled drops/drinking water) or indirectly (aerosols transmitted through the air due to the activity of handpieces) [16]. Microorganisms transmitted through aerosols can not only cause diseases such as flu and colds, but can also lead to respiratory diseases such as tuberculosis and Legionnaires' disease. Therefore, both the room where the dental treatments are performed and the dental service personnel and patients can be infected with the microorganisms inside these tubes [17–19].

A wide spectrum of microorganisms dwell in the human oral cavity. Over 250 species from the oral cavity have been isolated in culture and characterized, including several key pathogens, [such *as Entamoeba gingivalis, Streptococcus mutans, Porphyromonas gingivalis, Tannerella forsythia, and Aggregatibacter actinomycetemcomitans*] involved in the etiology of dental caries and periodontal disease [20–23]. Although oral microbiome plays an important role in incidence of oral diseases, but it has been detected in many distant organ sites too. Early research demonstrates that oral cavity-associated microbes can influence immune responses and disease pathogenesis outside the oral cavity [24]. The oral microbial ecosystem is constantly exposed to exogenous foreign substances and can be affected and changed by these exogenous factors [25].

Today, a large number of people in the community, including patients and staff of dental offices and clinics, are at risk of various infections due to dental treatment procedures and exposure to contaminated water particles in dental units. Also, the review studies conducted on this subject do not contain enough information regarding the prevalence of bacterial biofilm contamination of DUWLs and its frequency. Therefore, since the prevalence of biofilm contamination varies in different countries and to the best of our knowledge, there is no comprehensive study to summarize them, this review study aims to determining the prevalence of bacterial biofilms contamination in DUWLs.

## Methods

### Study design

This systematic review and meta-analysis were conducted according to the standard guideline of “*Preferred Reporting Items for Systematic Reviews and Meta-Analyses* (PRISMA)” [26].

### Search strategy

To find the related articles, a search strategy was planned, using different related keywords in different international databases, including *Medline* (via *PubMed*) and *Scopus*. The combination of *Medical Subject Heading* (MeSH) and selected keywords were used to search in *PubMed*. The search was filtered to English language studies without date restriction. The adapted keywords were used to search in Scopus database. The details of the search in *PubMed* and *Scopus* are briefly shown in Table 1. The search was performed by two authors (AAH, KR) on December 2021. Finally, to find the gray literature, *Google Scholar* was searched, and references of included studies were checked manually to find the more relevant studies.

**Table 1:** The search strategy in PubMed and Scopus databases

Search	Query
PubMed	("Biofilms"[MeSH Terms] OR "Biofilms"[Text Word] OR "Microbial Biofilm"[Text Word] OR "Bacterial Biofilm"[Text Word] OR "Fungal Biofilm"[Text Word] OR "Contamination"[Text Word] OR "Microbial Contamination"[Text Word] OR "Containment of Biohazards"[MeSH Terms]) AND ((("Waterline"[Text Word] OR "Water line"[Text Word] OR "Waterlines"[Text Word] OR "Water lines"[Text Word]) AND ("Dental"[Text Word] OR "Dental Chair"[Text Word] OR "Dental unit"[Text Word])) OR ("DUWL"[Text Word] OR "DUWLs"[Text Word] OR "DUW"[Text Word]))
Scopus	( ( ( ( TITLE-ABS-KEY ( dental) OR TITLE-ABS-KEY ( “Dental Chair”) OR TITLE-ABS-KEY ( “Dental unit”))) AND ( ( TITLE-ABS-KEY ( waterline) OR TITLE-ABS-KEY ( “Water line”) OR TITLE-ABS-KEY ( waterlines) OR TITLE-ABS-KEY ( “Water lines”)))) OR ( ( TITLE-ABS-KEY ( duwl) OR TITLE-ABS-KEY ( duwls) OR TITLE-ABS-KEY ( duw)))) AND ( ( TITLE-ABS-KEY ( biofilms) OR TITLE-ABS-KEY ( “Microbial Biofilm”) OR TITLE-ABS-KEY ( “Bacterial Biofilm”) OR TITLE-ABS-KEY ( “Fungal Biofilm”) OR TITLE-ABS-KEY ( contamination) OR TITLE-ABS-KEY ( “Microbial Contamination”)))

### Inclusion and exclusion criteria

Studies that have examined the prevalence of biofilms and contamination of water from different waterways of the dental unit, all studies that calculated the prevalence, studies that had English Full Text and were published in journals and even congresses were included in the meta-analysis. There was no restriction on the time of the study or the geographical location of the study. In-vitro studies, studies that did not have relevant data or used unusual laboratory methods and studies where the full text of the article was not available in English were excluded.

### Study selection

*Endnote* version X8 was used to screen the retrieved articles and to find the relevant studies. At the first, duplicate studies were removed and then, screening was done by titles and abstracts of the remaining articles and the studies that did not meet the inclusion criteria were excluded. After that, the screening was done based on full-text of remained articles and again, the studies that did not meet the inclusion criteria were excluded. The needed data was extracted from the obtained related articles. Screening of articles and extraction of required data were performed by two researchers independently and in case of disagreement, decisions were made in consultation with another author.

### Standard methods to measuring the bacterial contamination of DUWLs

There are different standards for measuring the bacterial contamination of DUWLs. In this study, three standard methods were used, including the method of the Centers for Disease Control and Prevention (CDC, more than 500 Colony Forming Unit/ milliliter (CFU/ml)), the American Dental Association (ADA, more than 200 CFU/ml) and the European union (EU) standard, whose criterion is more than 100 CFU/ml (C100). Each of the included studies may have measured and reported the prevalence of bacterial biofilm contamination of DUWLs using all three methods, or may have reported only one or two methods [27, 28].

### Data extraction

The required data were extracted from the full-text of retrieved articles by the authors independently (KR, SHM), and decisions were made in consultation with co-authors (MB, AAH) in the cases of disagreements. Data extracted from studies includes: first author’s name, year of publication, total number of samples, number of contaminated samples based on different standards, prevalence of biofilms (and its *95% confidence interval* (95% CI)), country, the methodological quality score of the studies, number of samples infected with Pseudomonas aeruginosa and number of samples infected with Legionella pneumophila were extracted.

### Risk of bias

The methodological quality of the articles assessed by the checklist was taken from a systematic review study conducted by Bain et al. In 2014 in the field of water pollution [29]. This checklist was adapted to evaluate the methodological quality of included studies in our meta-analysis (Additional file 1: Appendix 1). The score range of this checklist is from zero to nine. Finally, the articles were categorized in terms of quality and were placed in three levels with low quality with a score of less than three, medium quality with a score between three and six and high quality with a score of more than six.

### Statistical analysis

*Chi square* test and *I-square* statistic were used to evaluate the heterogeneity between articles and *I-square* above 50% was considered as heterogeneity. In analyzes where there was significant heterogeneity, the random-effects model was used and in cases where heterogeneity was not significant, the fixed-effect model was used. To check the publication bias, *Egger’s linear regression* (with “*metabias*” command [30]), was used. Because in some studies, the prevalence of bacterial contamination was reported to be 100%, conventional methods and packages were not able to calculate the confidence interval for these cases and excluded them from the meta-analysis. Therefore, to pool the results of different studies in this study, the *Metapreg* statistical package [31] designed for these cases was used. *Agresti-Coull* method was used to estimate the confidence interval. All statistical analysis were performed by *Stata* software version 16 (Stata Corp, College Station, TX, USA).

## Results

### Study selection and characterization

**Fig. 1:**
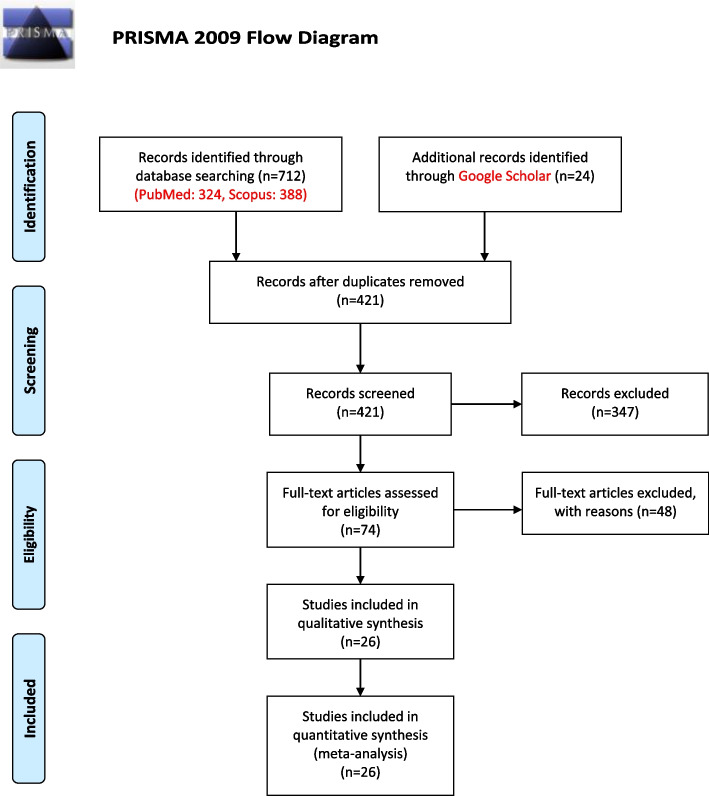
Flow diagram of the literature search for studies included in meta-analysis

As shown in Fig. 1, after updating the searches and the manual search in the *Google Scholar* database, a total of 736 studies (388 studies in *Scopus*, 324 studies in *PubMed* and 24 studies in *Google Scholar*) were identified and entered the *EndNote* 7 software for more precise investigation. Duplicate studies including 315 studies were eliminated and 421 studies remained for screening. In the screening phase, the titles of the studies along with the abstract were reviewed independently by two researchers, and studies that did not meet the inclusion criteria were excluded and disagreement cases resolved by third person. At the end of this phase, the full text of 74 articles were examined in more detail, and after removing irrelevant studies, 26 studies [15, 32–56] remained and included in the meta-analysis.

The number of samples in the studies varied from a minimum of 12 samples to a maximum of 717 samples, including samples of high-speed handpieces, air/water syringes, scalers, and so on. The average level of contamination also ranged from about 36 to more than 270,000,000 CFU per milliliter of water discharged from the unit's waterline. The oldest study was in 1976 and the most recent study was published in 2020. A summary of the study information is provided in Table 2.

**Table 2:** A summary of the included study characteristics

Year	Country	Author	Sample size	Number of positive samples			Pseudomonas Aeruginosa		Legionella pneumophila		Risk Score	Ref
				ADA	CDC	C100	Sample No	Positive	Sample No	Positive		
1976	USA	Gross A	57	38	33						2	[32]
1993	USA	Williams JF	182		131						3	[33]
2000	United Kingdom	Walker JT	110	91		104					7	[34]
2002	USA	Cobb CM	12	12	12	12					5	[35]
2003	Brazil	Souza-Gugelmin MC	30	26	26	27					4	[36]
2004	Spain	Araujo R	80	17							7	[37]
2004	European countries	Walker JT	474	242			474	18	474	5	8	[38]
2004	United Kingdom	Walker RJ	60	45	45	49					6	[39]
2008	Turkey	Goksay D	59	57	49	59	59	14	59	0	5	[40]
2009	Sweden	Dahlen G	405	283	263	302	405	0	405	61	4	[41]
2009	Iran	Nikaeen M	50	45	43						5	[42]
2009	Turkey	Turetgen I	123	112							6	[43]
2012	Italy	Pasquarella C	717		414		148	31	150	22	5	[44]
2012	Malaysia	Siang MM	75	21			75	9	75	0	4	[45]
2014	Iran	Dobaradaran S	260	260		260					9	[46]
2014	Turkey	Gungor ND	50	37			50	3	50	0	6	[47]
2014	Turkey	Kadaifciler DG	20	14	11						6	[48]
2014	Brazil	Libosa GM	18	18	16						4	[49]
2015	Italy	Leoni E	136	32					136	32	8	[50]
2018	India	Lal B	44	43							6	[51]
2018	China	Zhang Y	33		33	33					5	[52]
2019	Egypt	Gawish S	120				120	9			8	[15]
2019	China	Ji XY	611		268	320					9	[53]
2019	Italy	Spagnolo AM	30			30			60	47	7	[54]
2019	Netherlands	Volgenant C	214			59					5	[55]
2020	Ecuador/Venezuela	Castellano Realpe OJ	143		105		43	22			7	[56]

### Risk of bias within studies

Based on the modified checklist, the quality of the studies was evaluated, and the results are shown in Table 2. After categorizing the methodological quality score, there were 2 low quality, 15 moderate quality and 7 high quality studies.

### Quantitative data synthesis

#### Prevalence of bacterial contamination in samples according to ADA standard

**Fig. 2:**
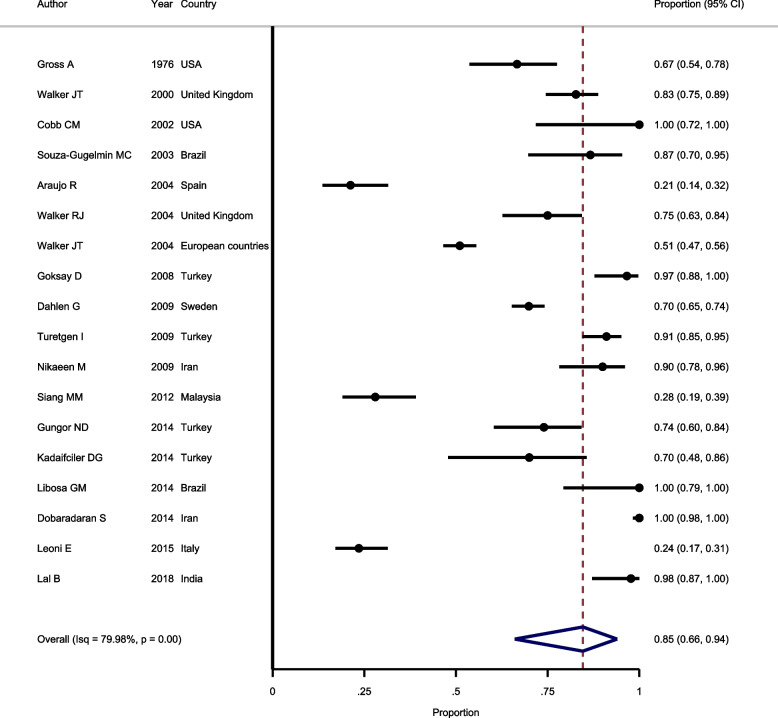
Forest plot showing the prevalence of bacterial contamination in samples according to ADA standard

To pool the prevalence of bacterial contamination in samples according to ADA standard 18 studies were included in the meta-analysis. Heterogeneity between articles was assessed using I-Square statistics. The results showed significant heterogeneity between included studies (I^2^ = 79.98%, Chi-squared = 534.2, *P* > 0.001). Because of significant heterogeneity, random-effect model was used to pool the results. As shown in Fig. 2, the pooled prevalence of bacterial contamination in samples according to ADA standard is estimated as 85.0% (*95%CI*: 66.0–94.0%). In assessing the publication bias, the result of Egger test does not show any evidence of publication bias (*P* = 0.150).

#### Prevalence of bacterial contamination in samples according to CDC standard

**Fig. 3:**
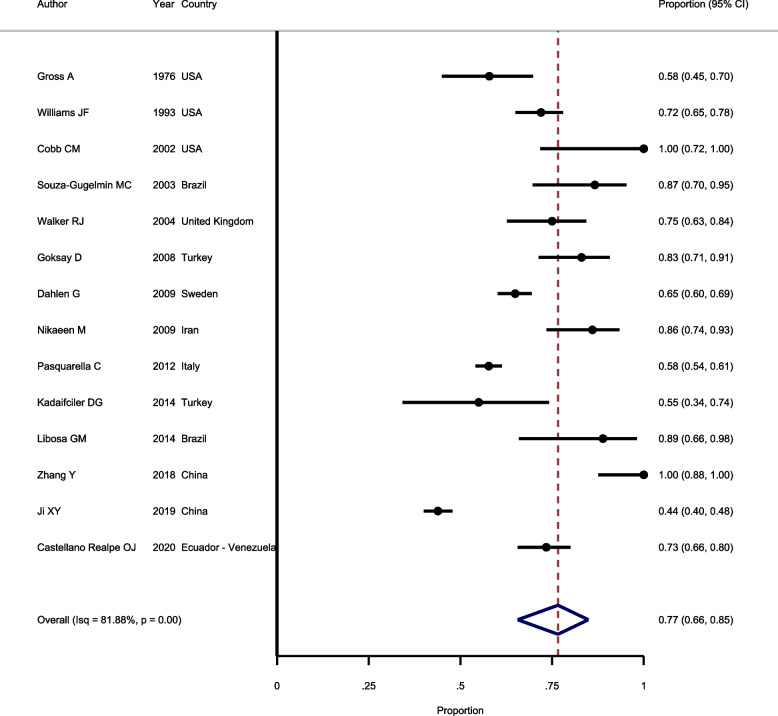
Forest plot showing the prevalence of bacterial contamination in samples according to CDC standard

The pooled prevalence of bacterial contamination according to CDC standard estimated by combining the results of 14 studies. In terms of heterogeneity, the results showed significant heterogeneity between included studies (I^2^ = 81.88%, Chi-squared = 140.6, *P* > 0.001). Because of significant heterogeneity, random-effect model was used to pool the results. As shown in Fig. 3, the pooled prevalence of bacterial contamination according to CDC standard is estimated as 77.0% (*95%CI*: 66.0–85.0%). Regarding publication bias, the results of the Egger test showed that studies have evidence of publication bias (*P* = 0.023).

#### Prevalence of bacterial contamination in samples according to C100 standard

**Fig. 4:**
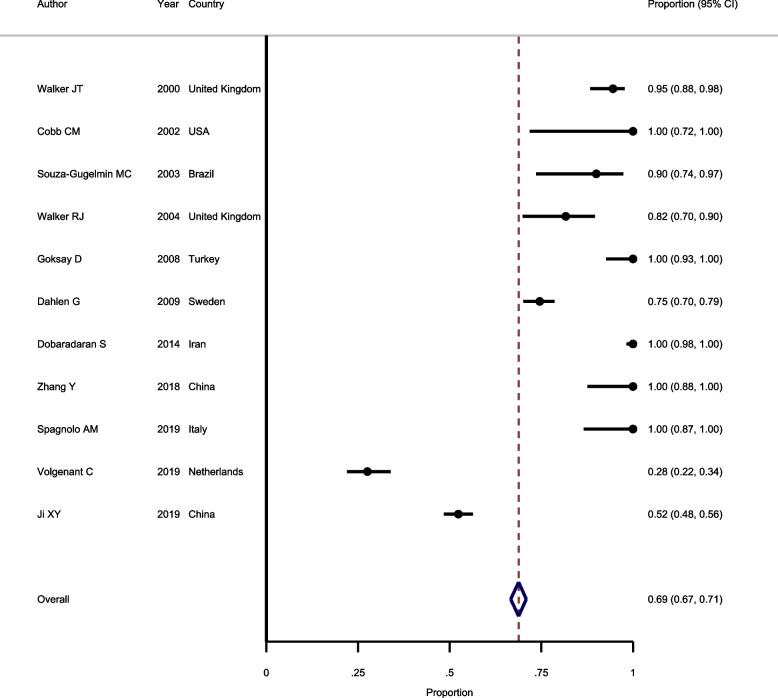
Forest plot showing the prevalence of bacterial contamination in samples according to C100 standard

Eleven studies were included in the meta-analysis to estimate the pooled prevalence of bacterial contamination according to C100 standard. In terms of heterogeneity, the results revealed that there is no evidence of heterogeneity between included studies (I^2^ = 12.9%, *Chi-squared* = 538.4, *P* > 0.001), therefore, fixed-effect model was used to pool the results. As shown in Fig. 4, the polled prevalence of bacterial contamination according to C100 standard is estimated as 69.0% (*95%CI*: 67.0–71.0%). Regarding publication bias, the results of the *Egger* test did not support the presence of publication bias (*P* = 0.647).

### Prevalence of Legionella pneumophila in waterlines

**Fig. 5:**
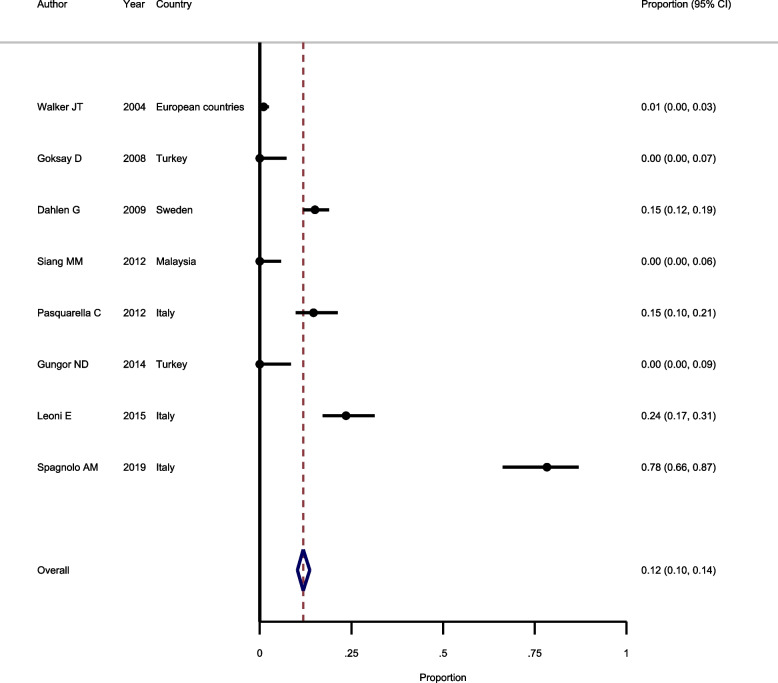
Forest plot showing the prevalence of Legionella pneumophila in waterlines

Eight studies were included in the meta-analysis to estimate the prevalence of *Legionella pneumophila*. Based on Chi square test, there is no heterogeneity between included studies and fixed-effect model was used to pool the results (I^2^ = 30.5%, *Chi-squared* = 255.7, *P* < 0.001). The results of fixed-effect model revealed that the overall prevalence of Legionella pneumophila is 12.0% (*95%CI*: 10.0–14.0%) (Fig. 5). The results of *Egger* test showed that there is no evidence in favor of publication bias (*P* = 0.189).

### Prevalence of Pseudomonas Aeruginosa in waterlines

**Fig. 6:**
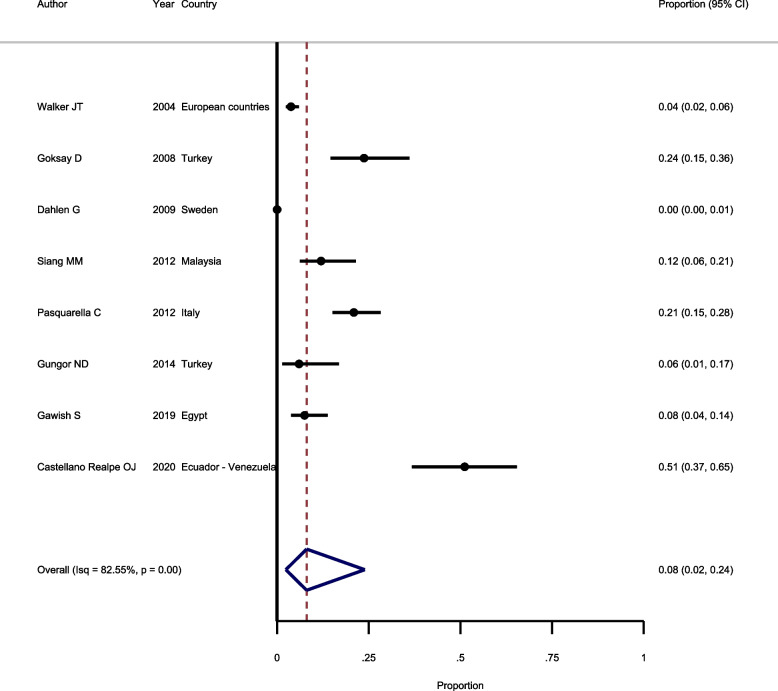
Forest plot showing the prevalence of Pseudomonas Aeruginosa in waterlines

Eight studies were included in the meta-analysis to estimate the prevalence of *Pseudomonas Aeruginosa.* Based on Chi square test, there is a significant heterogeneity between included studies and random-effect model was used to pool the results (I^2^ = 82.55%, *Chi-squared* = 141.0, *P* < 0.001). The results of random-effect model revealed that the overall prevalence of Pseudomonas Aeruginosa is 8.0% (*95%CI*: 2.0–24.0%) (Fig. 6). Based in *Egger* test, there is a publication bias (*P* = 0.038) among the included studies.

## Discussion

Twenty-six studies were eligible to be included in this meta-analysis. The main results of this meta-analysis suggested a relatively high prevalence of bacterial contamination in DUWLs. According to the ADA, CDC, and C-100 standards, the prevalence of bacterial contamination was estimated to be 85.0%, 77.0%, and 96.0%, respectively. The prevalence of *Legionella Pneumophila* and *Pseudomonas Aeruginosa* in DUWLs was estimated to be 12.0% and 8.0%, respectively.

There are many different ways to disinfect the DUWLs, such as use of Hydrogen peroxide, Chlorine dioxide, Chlorhexidine etc. [57–59]. Based on the latest systematic review on this subject, independent water reservoirs are recommended for disinfecting DUWLs using distilled water. Flushing DUWLs should be combined with disinfections. Nearly all the chlorine-, chlorhexidine- and peroxide-containing disinfectants, mouthrinses and citrus botanical extract meet the standard for disinfecting DUWLs. Alkaline peroxide would lead to tube blockage in the DUWLs. Regularly changing disinfectants can reduce the risk of occurrence of disinfectant-resistant strains of microbes [60]. Though every dental unit manufacturer has its own recommendations for DUWLs disinfection and clinicians can follow the manufacturer guideline for the proper waterline disinfecting.

DUWLs are highly prone to contamination by various microbial species. Because long-term accumulation of microbes in these lines can lead to the formation of biofilms, these waterlines, as a potential source of microorganisms, have the ability to spread contamination through aerosols produced during dental procedures. For this reason, the main concern is about the opportunistic pathogens that can infect people with suppressed immune systems and, through cross-infections, pose a risk to their health as well as to dental staff who are exposed to these contaminants on a daily basis.

According to the three standard methods (ADA, CDC and C100), the minimum estimated prevalence of bacterial contamination was 69% in our meta-analysis. In the study of Spagnolo et al. [54] in Italy, the contamination in all water samples (*n* = 30) of dental units exceeded the EU standard (EU drinking water standards/ more than 100 CFU/ml). Walker et al. [38] investigated 474 samples of waterlines in seven European countries (Britain, Ireland, Greece, Spain, Germany, Denmark, and the Netherlands) and found that 51% of the samples were contaminated more than the limit set by the ADA (more than 200 CFU/ml). Also, approximately 20% of the air–water syringe and handpiece samples in Greece and Ireland showed evidence of non-hemolyzed blood [38].

The study by Ji XY et al. [53] in China between 2012 and 2017 was one of the most comprehensive studies in the field of contaminated water from units, with a total of 611 samples from 318 units in 64 clinics over the 6-year study period. The prevalence of contamination based on CDC standard (more than 500 CFU/ml) was estimated to be close to 44%. One of the interesting points in this study was that for nearly 80% of the samples, no action had been taken to disinfect the waterlines before the study.

Another study was conducted by Pasquarella et al. [44] in nine Italian cities and ten clinics, of which seven clinics were educational. Of the 717 samples taken, more than 57% were contaminated according to CDC standards. Other important investigation performed in this study includes sampling of waterlines before and after clinical activity. Due to the water circulation in the waterlines during the activity and working with the unit, the average level of contamination in the water leaving the waterlines after clinical activity was statistically significantly lower than that before the activity.

The results of this meta-analysis showed that the prevalence of bacterial contamination in waterlines of dental units is very high. According to the ADA standard, the prevalence of bacterial contamination was reported between 21 and 100%, and the results of the meta-analysis were 85%. That is, 85% of the dental units were contaminated according to the ADA standard. According to the CDC standard, it ranged from 44 till 100% and the results of meta-analysis was 77%. Finally, based on C-100 standard, the pooled prevalence of bacterial contamination was 69% and it is varied from 28 to 100% in the included studies. All in all, it can be concluded that according to all three standards, the prevalence of bacterial contamination is very high, and it is recommended to review the design of the units and the correct and timely use of disinfection protocols.

In a series of studies conducted in this field, various factors, such as sampling from the private or public sector, disinfection protocols, age of units, routine services provided to units, and many other factors, lead to a high heterogeneity of statistical results reported in different studies. A number of studies have investigated the effect of flushing on reducing the load of contamination in waterlines [61, 62]. Flushing is a method of reducing the contamination load in waterlines by circulating water through the unit hoses [62]. The final result of a study conducted by Alkhulaifi et al. in 2020 in Saudi Arabia, showed that the amount of bacterial contamination after flushing for 3 min was significantly reduced compared to the contamination load of the prototype. However, flushing leads to reduce the microbial load, but it will not reduce the microbial load below the standard level in DUWLs that have non-standard pollution [61]. A study by Cobb et al. also showed that flushing, even for 2–4 min, could significantly reduce the number of planktonic bacteria in the water leaving the unit [35].

However, in a study by Türetgen et al. [43] in Turkey, to compare the contamination of incoming and outgoing water from units, 21 units in the private sector and 20 units in the government sector were sampled. In the case studies, it was reported that the age of the units varied from 3 months to 35 years, but no statistically significant relationship was found between the age of the unit and the amount of contamination of the unit's outgoing water.

Also, in some studies, bacterial species and strains present in the water systems of the units were identified by culture, DNA sequencing methods, and by studying the amount of bacterial endotoxin [52, 61, 63–65]. In the study by Zhang Y [52] on DNA sequencing of bacteria presented in the outgoing water of the units, 9 lineages and 42 bacterial genera were identified. Also, during sequencing in this study, a large number of non-culturable bacteria were found through conventional methods. Pathogenic and opportunistic bacteria found in this study include *Legionella, Pseudomonas*, *Acinetobacter, Methylophilus, Escherichia-Shigella, Streptococcus,* and *Flavobacterium*.

Among all the identified pathogens, *P. aeruginosa* and *L. pneumophila* are the main pathogens that have been studied in a number of studies. The results of our meta-analysis revealed that the prevalence of *Legionella pneumophila* and *Pseudomonas aeruginosa* in DUWLs was estimated to be 12.0% and 8.0%, respectively.

The following are the limitations of this study. In this study, we searched only two databases including *PubMed* and *Scopus*, and only studies with English full-text were included in this meta-analysis. In some cases, even though the study was relevant, the required data was not reported, and despite contacting the authors of the articles, no response was received and they were inevitably excluded from the study. One of the strengths of this study is the summary of all published articles in this field.

## Conclusion

The results of this review study suggested a high prevalence of bacterial biofilm prevalence in DUWLs; therefore, the use of appropriate disinfectants is recommended to reduce the prevalence of contamination and reduce the infection. Due to the high prevalence of bacterial contamination in this setting, it is recommended to use different disinfectants and sterilization to control contamination such as ozonisation, ventilation, ionization, fogging and UV light.

## Supplementary Information

**Additional file 1:** **Appendix 1.** Criteria to assess study quality.

## Data Availability

All data generated or analyzed during this study are included in the article.

## Abbreviations

ADA: American Dental Association
CDC: The Center for Disease Control and Prevention
DUWLs: Dental Unit Water Lines
CFU: Colony Forming Unit
CI: Confidence Interval
MeSH: Medical Subject Headings
PRISMA: Preferred Reporting Items for Systematic Review and Meta-Analysis
