# Supplementary material for: Bacterial biofilm prevalence in dental unit waterlines: a systematic review and meta-analysis
Source: BMC Oral Health. 2023 Mar 18;23:158. doi: 10.1186/s12903-023-02885-4 (PMC10024400; doi:10.1186/s12903-023-02885-4)
Supplement: Supplementary file 1 — Additional file 1: Appendix 1. Criteria to assess study quality. [file 12903_2023_2885_MOESM1_ESM.docx]

## Criteria to assess study quality (Adapted from Bain et al. 2014)

| Criteria | Description |
| --- | --- |
| Selection representative | Did the authors detail an approach designed to provide representative picture water quality in a given area? |
| Selection randomized | Was sampling randomized over a given study area or population? |
| Quality control | Were quality control of dental unit waterline procedures specified or referred to? |
| Method described | Are well-defined and appropriate methods of microbial analysis described or referenced? |
| Point of sampling | Was the point at which water was sampled well defined? (For example, whether the water was collected from an air/water syringe, highspeed handpiece, etc.?) |
| Handling described | Are sample handling procedures described, including sample collection, transport method, and duration? |
| Handling minimum criteria | Does sample handling and processing meet the following criteria: transport in cool box between 2-6°C, analysis within 6 hours of collection, and specified incubation temperature? |
| Method described | Clear explanation of the analytical method used to count microbial contamination and diversion or to calculate statistical analysis. |
| External review | Was the study subject to peer review or external review prior to publication? |
